# Science Is at a Crossroads—and Physiology With It: A Statement from the APS Chief Executive Officer

**DOI:** 10.1093/function/zqaf025

**Published:** 2025-06-09

**Authors:** Scott Steen

**Affiliations:** Chief Executive Officer, American Physiological Society, Rockville, MD 20852-9839, USA

We are living through a moment of unprecedented challenge for science in the United States. A few months ago, as the Chief Executive Officer (CEO) of the American Physiological Society (APS), I had the opportunity to address our community of biomedical researchers, educators, and innovators at the opening session of our annual summit. It was a gathering not only of peers, but of people united by a shared commitment to discovery, health, and the public good. And it happened at a time that feels markedly different from those that have come before.

The atmosphere surrounding science, academia, and trust in expertise is shifting—and not for the better. We are facing intensified scrutiny, shrinking funding, and the politicization of science. Public health infrastructure is being undermined just as we continue to reckon with the lessons of a global pandemic. Research institutions are under enormous pressure, and misinformation is not just spreading—it is being rewarded.

These threats are not abstract. They are existential, challenging the very core of the scientific enterprise.

There is a growing effort to devalue expertise, to sow distrust in peer review, to question the motives of researchers, and to dismiss the slow, collaborative process of discovery as elitist or irrelevant. Science is being rebranded—not as a public good, but as a partisan tool. Regardless of personal politics, that should alarm every one of us.

We are witnessing erosion across the spectrum—from the silencing of government scientists to cuts in critical research programs, to new barriers to international collaboration, student visas, and the free exchange of ideas. Words that reflect the diversity of our nation and the scope of our scientific inquiries are being erased from government websites. At some institutions, entire programs related to climate science, reproductive health, or pandemic preparedness are being eliminated or quietly buried.

This is not just about budgets or bureaucracies. It’s about the kind of society we want to live in—and the kind of future we are building for the next generation of scientists.

For many in our community, these threats are not only professional—they are deeply personal.

Members of our community from historically underrepresented backgrounds are carrying a dual burden: the persistent challenges of equity in science, now compounded by a rising tide of hostility. Initiatives to expand opportunity, foster inclusion, and build belonging are being dismantled in real time. Faculty are being silenced. Grants supporting critical health research—especially work focused on women, racial and ethnic minorities, and LGBTQ people—are being canceled, often without scientific justification.

This is not merely a policy debate—it’s a moral one. We know that disease does not affect all groups equally. A scientific community that fails to reflect the diversity of our society is not only unjust, but also incomplete. Innovation thrives when a broad range of experiences and perspectives come to the table.

You don’t need to look far to see the scale of the threat. Scientific consensus is being undermined—not by data or new insight, but by political forces and public voices unmoored from evidence. Conspiracy theories and ideology are replacing experimentation and peer-reviewed research. At the same time, federal support for biomedical research is being slashed—often abruptly and without explanation. Indirect costs are being gutted. The infrastructure of discovery is being deliberately weakened.

This is not the moment for silence or neutrality. It is a moment for clarity, for solidarity, and for resolve. We owe that to our students. We owe it to the patients waiting for cures. We owe it to the public, whose health depends on a scientific enterprise they can trust. And we owe it to ourselves.

In the face of these challenges, APS is responding with resolve and vision.

We are doubling down on our commitment to scientific excellence. We’ve convened a task force of some of the nation’s most distinguished scientists—including Nobel Laureates and National Academy members—to help define and elevate standards of excellence across our organization. Their recommendations will inform how we engage with and grow the community of researchers shaping the future of physiology.

Our publishing strategy reflects this same commitment. Through our new Subscribe to Open). model, all 10 participating APS journals met their participation goals in the first year—making more physiology content freely accessible without added cost to authors. We are also investing in the full potential of *Function* as the leading research journal for the discipline, just as *Physiological Reviews* is our flagship for scholarly synthesis.

But publishing is not enough. We also must dramatically step up our advocacy for, and defense of, both the discipline of physiology and science itself.

Over just the past few months, APS members have sent more than 2 500 messages to Congress in support of science funding. During the summit, 70 members—including our board—visited Capitol Hill to deliver the message that science is essential. We will be expanding these efforts, both independently and through strategic alliances, to protect scientific research, academic freedom, and our shared values.

We are also launching one of the most ambitious public outreach efforts in APS history. With the support of our board, we’re partnering with a top-tier, scientific public affairs firm to reshape how physiology is perceived across policy, funding, and public spheres. This campaign will include media outreach, digital strategy, and compelling storytelling designed to reintroduce physiology to the broader scientific ecosystem and the informed public.

We do this not for attention, but because physiology and biomedical research are indispensable—and this is the moment to prove it.

I am not a scientist. But I have spent much of my career working alongside scientists—supporting their work, learning from their passion, and doing everything I can to sustain the ecosystems that make discovery possible. And I can tell you this: I have never seen a moment quite like this one.

But we must not be defined by the forces working against us. We must be defined by how we respond.

Now is the time to act—with focus, urgency, and unity. Every one of us has a role to play, whether at the bench, in the classroom, or through advocacy and leadership. And APS is here to support that work.

Years from now, I believe we will look back and say: This was the inflection point. This was when we chose to meet fear with integrity. When we stood up for science, for truth, and for each other. When we built something stronger—not in spite of the pressure, but because of it.

That is the legacy we need to build together.

